# Transduction of ferret airway epithelia using a pre-treatment and lentiviral gene vector

**DOI:** 10.1186/1471-2466-14-183

**Published:** 2014-11-21

**Authors:** Patricia Cmielewski, Nigel Farrow, Martin Donnelley, Chantelle McIntyre, Jahan Penny-Dimri, Tim Kuchel, David Parsons

**Affiliations:** Respiratory and Sleep Medicine, Women’s and Children’s Hospital, 72 King William Road, North Adelaide, SA 5006 Australia; Centre for Stem Cell Research, University of Adelaide, Adelaide, SA 5001 Australia; School of Paediatrics and Reproductive Health, University of Adelaide, Adelaide, SA 5001 Australia; South Australian Health and Medical Research Institute, Gilles Plains, SA 5086 Australia

**Keywords:** Ferret, Lung, Lentivirus, Cystic fibrosis, Gene therapy

## Abstract

**Background:**

The safety and efficiency of gene therapies for cystic fibrosis (CF) need to be assessed in pre-clinical models. Using the normal ferret, this study sought to determine whether ferret airway epithelia could be transduced with a lysophosphatidylcholine (LPC) pre-treatment followed by a VSV-G pseudotyped HIV-1 based lentiviral (LV) vector, in preparation for future studies in CF ferrets.

**Methods:**

Six normal ferrets (7 -8 weeks old) were treated with a 150 μL LPC pre-treatment, followed one hour later by a 500 μL LV vector dose containing the LacZ transgene. LacZ gene expression in the conducting airways and lung was assessed by X-gal staining after 7 days. The presence of transduction in the lung, as well as off-target transduction in the liver, spleen and gonads, were assessed by qPCR. The levels of LV vector p24 protein bio-distribution in blood sera were assessed by ELISA at 0, 1, 3, 5 and 7 days.

**Results:**

The dosing protocol was well tolerated. LacZ gene expression was observed *en face* in the trachea of all animals. Histology showed that ciliated and basal cells were transduced in the trachea, with rare LacZ transduced single cells noted in lung. p24 levels was not detectable in the sera of 5 of the 6 animals. The LacZ gene was not detected in the lung tissue and no off-target transduction was detected by qPCR.

**Conclusions:**

This study shows that ferret airway epithelia are transducible using our unique two-step pre-treatment and LV vector dosing protocol. We have identified a number of unusual anatomical factors that are likely to influence the level of transduction that can be achieved in ferret airways. The ability to transduce ferret airway epithelium is a promising step towards therapeutic LV-CFTR testing in a CF ferret model.

## Background

Cystic Fibrosis (CF) is an autosomal recessive disorder caused by a mutational error in the Cystic Fibrosis Transmembrane Conductance Regulator (CFTR) gene and its associated protein [1]. The CFTR channel is used in multiple body systems that utilise epithelial ion gradients, including but not limited to the pancreas, sweat glands, gastrointestinal tract and most crucially the lungs [1]. The resultant dehydration of the airway surface, loss of cilia movement, and accumulation of highly viscous mucous obstructs the airways and hinders clearance, thereby promoting bacterial infection [2]. CF airway disease currently limits the life expectancy of a CF patient to a predicted median survival estimate of approximately 37 years [3]. Therefore, the successful treatment of CF airway disease, especially from the earliest stages of life, is imperative in improving short term and long term survival of those afflicted with CF.

Gene therapy for a loss-of-function disease like CF involves delivering the appropriate corrective DNA into the cells of an organism to produce adequate functional protein to ameliorate the symptoms of that disease. Lentiviral gene vectors have a number of potential benefits, including the ability to transduce both dividing and non-dividing cells, be pseudotyped with an envelope glycoprotein to allow for broad tissue tropism [4] and have the ability to carry large genes such as CFTR [5].

Amongst the new generation of CF animal models, the CFTR knockout ferret exhibits many characteristics homologous to human CF, including disease of the pancreas, liver, vas deferens; and variably-penetrant meconium ileus. The similarities between CF humans and CF ferrets in their respiratory pathophysiology include defective chloride transport, submucosal gland fluid secretion, and a propensity towards lung infection in the early post natal period [6].

The aim of this study was to expand work done in the mouse, sheep and marmoset [7–9] and test the ability of our two-step LPC pre-treatment and lentiviral vector delivery system to transduce airway cells of the adolescent normal ferret. The rationale for this work was to prepare for the use of CF ferrets, by establishing dosing, technical, and analytical procedures to be applicable for future use in these expensive and husbandry-intensive disease-specific CF animals.

## Methods

This study was approved by the Animal Ethics committees of the Women’s and Children’s Health Network, South Australia; SA Pathology, South Australia; and the University of Adelaide, South Australia. Six 7-8 week old adolescent ferrets *Mustela spp* (2 male and 4 female, weight 335 to 460 g) were sourced from a commercial ferret supplier. Experiments and subsequent monitoring were performed under specialist veterinarian supervision at the Gilles Plains South Australian Medical Research Facility.

### Gene vector

The nuclear-localised LacZ (LacZnlsco) VSV-G pseudotyped HIV-1 based LV vector was produced by transient transfection of 293T cells using a five plasmid system according to previously published protocols [9, 10]. The ratio of plasmids used for all virus preparation (per 245 mm square plate) was 170 μg of the transgene 1SDmMPSV LacZnlsco, 3.16 μg of pcDNA3 Tat, 3.16 μg of pHCMVRev, 1 μg pHCMVgagpol, 7.9 μg pHCMV-G.

For purification approximately 1 L of vector supernatant was collected, and passed through a 0.45 μm filter (Whatman Polydisc AS, GE Healthcare, PA, USA) and then two MustangQ Acrodiscs (Pall Corporation, NY, USA) connected in series, at a flow rate of 10 ml/min. After loading, the Acrodiscs were immediately flushed with 30 ml of PBS. The viral particles were then eluted with 4 ml of 1.5 M NaCl into a sterile tube containing an equal volume of 2% (v/v) mouse serum in H_2_O. The virus was concentrated by ultracentrifugation at 20,000 rpm (53,750 × g) for 90 minutes at 4°C and then resuspended in 6 × 100 μl aliquots of 0.9% (w/v) NaCl /0.1% (w/v) mouse serum albumin and stored at -80°C. A second batch was made in the same manner and added to the first without thawing.

Virus titre was 4.7 × 10^8^ TU/ml as assayed by qPCR [11]. The LV vector volume (500 μl) was established based on body weight linear scale-up from vector deliveries in mice [7] and comparable dosage protocols in other animal models, including marmoset and sheep [7, 8].

### *In vitro*assessment of vector delivery methods

Pre-treatment and vector were delivered using an ~15 cm PE delivery cannula (Sterihealth, VIC, Australia) attached to a syringe, to reach the distal portion of the very long ferret trachea (~9 cm with 60-70 cartilage rings [12]). To minimise delivery losses using the long cannula we tested several vector delivery methods in cell culture. Vector delivery was tested in a 10:1 scale system on CHO cells: (a) as a bolus delivered using a standard 200 μl lab pipette (control); (b) using the cannula and syringe setup; (c) via the cannula setup with a 20 μl air chaser [13]; (d) via the cannula with a 20 μl primer of 0.3% Bovine Serum Albumin (BSA, Sigma Aldrich, Cat # A7906), a known LV vector stabiliser. Note that only the 50 μl LV vector was delivered to the cells, leaving the 20 μl BSA in the syringe.

CHO cells were seeded at 0.5 × 10^6^ cells/ml in 12 well plates and incubated at 37°C, 5% CO_2_ for 3 hours in F12 Hams Media (SAFC Biosciences (USA) Cat # 51655)/10% (v/v) Fetal Calf Serum (FCS, JRH Biosciences (USA) Cat # 12103)/2 mM glutamine (SAFC Biosciences (USA) Cat # 59202C) and 1:1000 penicillin/streptomycin (pen-strep, Sigma Aldrich (USA) Cat # P4458). Media was aspirated, replaced with F12/10% FCS/glutamine/pen-strep supplemented with 4 μg/ml polybrene and 2 μg/ml gentamycin and cells were transduced with the LV-LacZ vector diluted 1:200. A 50 μl aliquot of the LV vector was added to wells in triplicate, using the four groups described above. Plates were incubated at 37°C and media was changed at 24 hours and replaced with F12/10% FCS/glutamine for a further 48 hours. Media was aspirated and cells were rinsed with PBS and fixed with 0.1% glutaraldehyde in PBS for 15 minutes on a rocking platform at room temperature. Cells were washed 3 times in 1 mM MgCl_2_/PBS for 10 minutes each and were incubated overnight with 1:40 dilution of Pre-Xgal (35 mM K_3_Fe(CN)_6_, 35 mM K_4_Fe(CN)_6_ 1 M MgCl_2_ : Xgal (40 mg/ml in dimethylformamamide)) solution at 37°C. The Xgal solution was aspirated, rinsed with PBS twice, and cells were stored in 80% glycerol. LacZ gene expression was quantified as the number of blue stained cells averaged from 3 fields of 3.83 cm^2^/well using light microscopy.

### Ferret *in vivo*pre-treatment and LV dosing

Anaesthesia was induced in each of the ferrets with a s.c. injection of medetomidine (Domitor, 0.15 mg/kg, Orion Corporation, Finland) and ketamine (12 mg/kg, Parnell Laboratories, Australia). Animals were intubated with an endotracheal tube (14 Ga. BD Insyte i.v. cannula, extended to 15 cm in length with polyethylene tubing) placed so that the tip was midway between the epiglottis and the carina. All dosing events were performed with the ferret held in a prone position, with the head raised at a 20 degree angle. The pre-treatment was a 150 μl of LPC (0.1% in PBS, Sigma Aldrich L4129). The concentration was derived from studies in mouse models [7, 14] and the volume was scaled up in the same manner as for the gene vector. The airway pre-treatment was administered through the PE cannula placed to project ~2 mm past the end of the endotracheal tube proximal to the carina, via a single bolus delivery over 10 seconds. Anaesthesia was maintained with a second s.c. injection of medetomidine (0.07 mg/kg) and ketamine (6 mg/kg) 30 minutes after pre-treatment. Lentiviral dosing was performed 1 hour after pre-treatment. Ferrets were kept intubated and maintained in a supine position for the hour after pre-treatment. A single priming dose of 200 μl BSA was drawn into the cannula, followed by the 500 μl LV-LacZ (200 μl diluted with 300 μl PBS) vector to be delivered. The 500 μl gene vector dose was administered through a cannula inserted through the endotracheal tube using a single 500 μl bolus delivery over 15 seconds. Animals were kept anaesthetised and prone for a further 20 minutes following dosing. Anaesthetic was reversed using an s.c. injection of atipamezole (Antisedan, 1.5 mg/kg, Orion Corporation, Finland). Vital signs were monitored during pre-treatment and dosing as well as in the post-operative period.

### Monitoring and tissue harvesting

Body weight and general behaviour were monitored daily, and blood samples were taken at baseline, 1, 3, 5 and 7 days after LV dosing. Samples were centrifuged at 13,000 rpm, with sera stored at -80°C. Animals were humanely killed one week after dosing by i.p. nembutal overdose (Lethabarb, >100 mg/kg). The right-most rostral lobe of the lungs was ligated and the remainder of the lungs was inflation-fixed *in situ* for 15 minutes in 2% paraformaldehyde/0.5% glutaraldehyde (PFA/Glut) in PBS at 4°C and a pressure of 30 cmH_2_O. The right-most rostral lobe of the lung was snap-frozen (dry ice), along with samples of liver, spleen and gonads.

### LacZ gene expression: Histology

The inflation fixed lung was then excised and submerged in fresh chilled PFA/Glut overnight, and were processed for LacZ expression by X-gal staining [7]. The extent of LacZ gene expression throughout the airways of the lung was assessed *prima facie* though examination of gross transversely-sectioned portions of trachea and lung. Portions that had indications of blue LacZ cell staining were prepared for routine histological sectioning and stained with a light eosin counterstain using standard methods.

### LV vector presence: p24 ELISA analysis of sera

Blood sera was analysed using an HIV-1 p24 ELISA kit (Perkin Elmer Life Sciences USA) performed as per manufacturer instructions.

### LacZ gene presence: qPCR

Tissue samples were processed to extract DNA via the Wizard SV Genomic DNA Purification System (Promega, USA, Cat. # A2361) as per manufacturer instructions.

Qualitative PCR (CFX Connect Real-Time PCR, Bio-Rad) was used to identify the presence of integrated NLS-LacZ gene, compared to the ferret GAPDH housekeeping gene [15]. PCR was performed in 8 well strips and specific amplification was detected using a TaqMan probe master mix according to the manufacturer’s standard protocol. All samples were performed in triplicate including a non-template control under the following cycles: 50°C for 2 min, 95°C for 10 min, 40 cycles of 95°C for 15 sec and 60°C for 1 min. Cycle thresholds (C_t_) for the LacZ gene were normalised with respect to the housekeeping gene (∆C_t_) and copy numbers per cell of the gene were determined from ∆Ct [8]. The following primers were used: NLS-LacZ forward GCC ACT TCT TGA TGG ACC ACT T, NLS-LacZ reverse CCG CCA CCG ACA TCA TCT, NLS-LacZ probe FAM-CAC GCG GGC GTA CAT-NFQ, GAPDH forward CAT CCG GTG TAC CTT TCC TT, GAPDH reverse CCA GGA AGA CAG GGA GAG TG, and GAPDH probe GCA CTG CTG CCA TGC (GeneWorks, Australia).

### Statistical analysis

Results are represented as a mean and standard error of the mean. Statistical analyses were performed using GraphPad Prism 6. Statistical significance was set at p = 0.05. Multiple treatment groups were analyzed by one way analysis of variance (ANOVA) with Dunnett’s multiple comparisons.

## Results

### *In vitro*assessment of vector delivery methods

**Figure 1:**
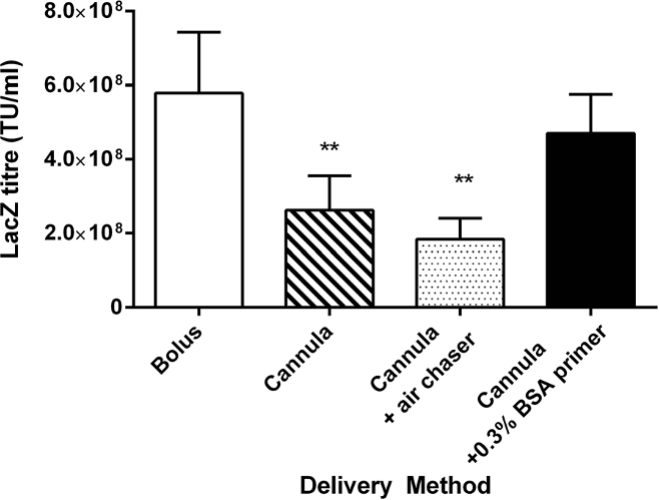
***In vitro*** **assessment of vector delivery methods.** *In vitro* testing showed significant reductions in titre when the vector was delivered via the 15 cm PE cannula, with or without an air chaser (**p <0.01, ANOVA compared to bolus, mean ± SE, n = 3). The addition of a BSA primer maintained a titre that was not significantly different to the bolus delivery, so this delivery method was chosen for all *in vivo* experiments.

Figure 1 shows that direct bolus delivery through the 15 cm cannula produced significant loss of vector titre compared to a bolus control (p <0.01, ANOVA). Furthermore, using an air chaser to maximise volume delivery also resulted in a significantly reduced titre. However, using a liquid BSA primer was not significantly different to the bolus delivery, so this method was used for all subsequent animal studies.

### Animal health

Based on vital signs monitoring and behavioural observations throughout the study the airway gene delivery procedure was well tolerated in all animals.

### LacZ gene expression: Histology

**Figure 2:**
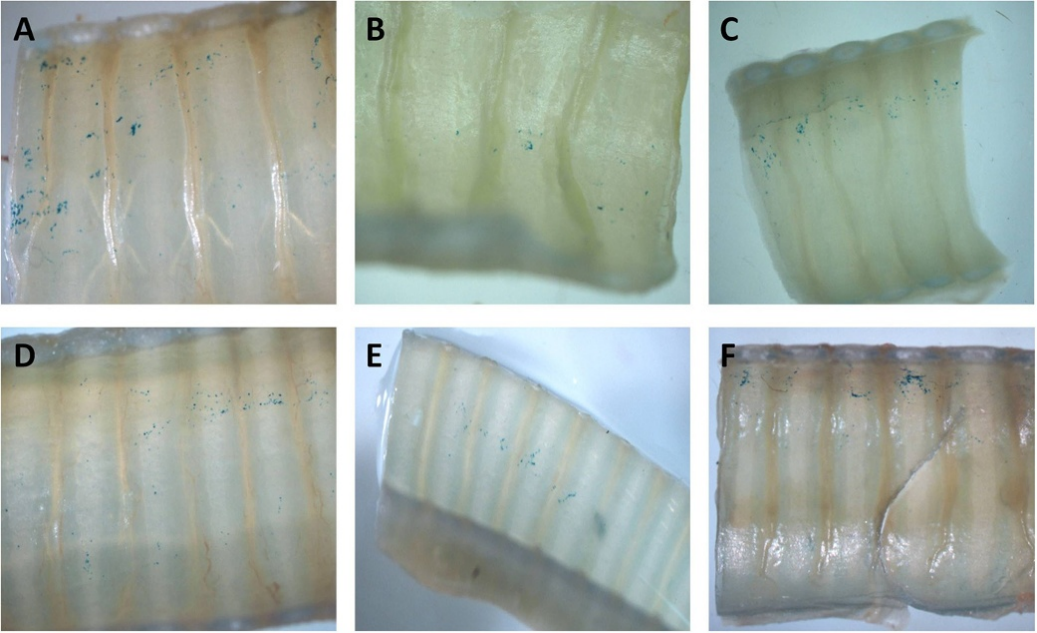
***En face*** **tracheal sections.** X-Gal stained *en face* sections of trachea (distal to the cannula tip) from all six ferrets **(A**-**F)**, following intra-tracheal delivery of 150 μl of 0.1% LPC followed one hour later by 500 μl LV-LacZ, demonstrate low levels of patchy LacZ gene transduction at 1 week (20× magnification).

**Figure 3:**
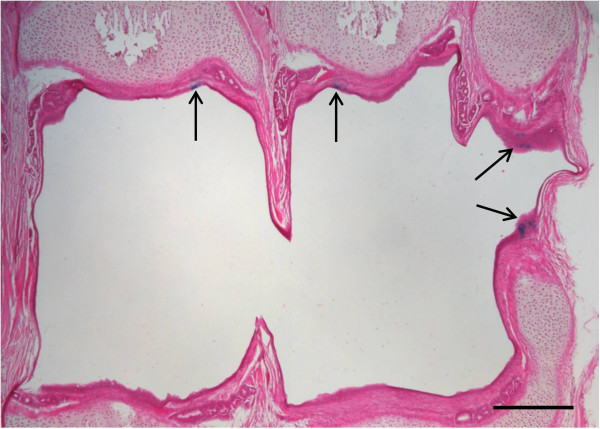
**Tracheal histology.** Light eosin-stained tracheal cross section of trachea shows patchy LacZ transduction. Scale bar 100 μm. Arrows mark LacZ transduced tracheal cells.

**Figure 4:**
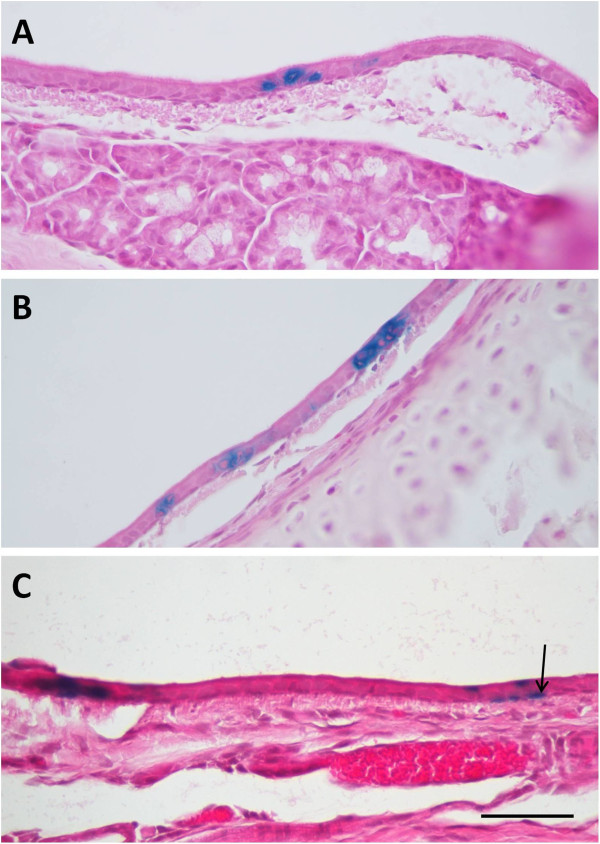
**High-power tracheal histology.** Light eosin-stained high-power sections of trachea show LacZ transduction of ciliated and basal cells **(A**-**C)**. Arrow in C points to an example of a transduced basal cell. Scale bar 10 μm.

**Figure 5:**
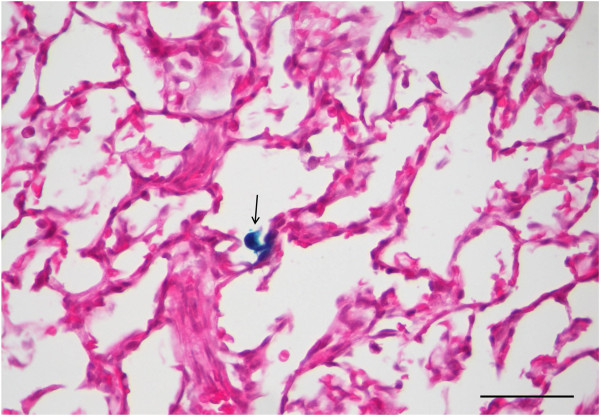
**Lung histology.** In the lung only rare LacZ-stained alveolar macrophages (arrow) were detected in one or two lobes, in some animals. Scale bar 10 μm.

The level of LacZ gene transfer was assessed *en face* and in cross-sections of ferret trachea and lung. All six ferrets showed small amounts of LacZ gene expression in the trachea, evident as blue-stained cells, with some stained cells present in the lobular regions of some animals. Figures 2, 3, 4 show typical examples of the patterns and extent of gene expression in the trachea, including both ciliated cells and basal cells. Areas of irregular lines or intense patches of blue-stained cell clusters were apparent. The majority of the LacZ expression was found in the trachea with very little extending into the lower lung airways and alveolar regions. Rare scattered LacZ transduced cells were observed in the lung (see Figure 5), but these were localised within the upper lobes rather than spread evenly across all lobes.

### LV vector presence: p24 ELISA analysis of sera

The presence of the p24 protein in the blood is an indication of vector or vector constituents that have passed from the airway into the vascular space after dosing. p24 protein in serum was above the threshold of detection in one animal at day 1. This animal also had the highest level of LacZ transduction, as assessed subjectively during *en face* examination.

### LacZ gene presence: qPCR

The presence of the LacZ gene in the lung, liver, spleen and gonads was assessed with qualitative PCR (qPCR). The LacZ gene was not detected in any of the animal tissues assayed.

## Discussion

Our *in vitro* findings were surprising, firstly due to the magnitude of the losses when using the long cannula, and secondly that the losses could be prevented using a BSA primer. Use of an air chaser – as used by other lung gene vector dosing studies to ensure complete volume delivery and wide distribution [13] – may not be an appropriate delivery strategy for use with LV vectors.

This study was designed to determine if the established LPC and gene vector delivery system we have developed in mice [7, 9, 11, 14, 16] – and has been ulitised by others in normal pigs [17] and rabbits [18] – would also transduce the conducting airway tissues of the normal ferret. The results show that the tracheal airway in normal ferrets is transducible using our lentiviral gene transfer system employing an LPC pre-treatment. While total LacZ gene expression was low (but greater than that achieved in sheep [7]) the patchy pattern of LacZ marker gene expression is similar to that observed in other animal models [7, 8, 14].

Importantly, histological examination showed transduction of both ciliated cells and basal cells. Transduction of these cell types is important, firstly because airway ciliated cells are a primary target for a CF gene therapy, with the aim to restore the ASL depth and allow normal mucocilliary clearance to be driven by properly functioning ciliated cells. Secondly, the ability to transduce respiratory basal stem cells enables the corrected CF gene to be passed on to all of its progeny during normal epithelial cell turnover, resulting in sustained gene expression.

There are a number of possible reasons why lower than expected levels of transgene expression were observed in this study: (1) the viral titre was a approximately 10-fold lower than previously used; (2) the lung volume of the ferret is not proportional to body size and exceeds the predicted value by 297% [19], so a linear volume scale up by weight from mouse may not be appropriate; (3) the trachea is longer than other similarly-sized animals (9 cm with 60-70 C-shaped tracheal rings [12]).

The upscaling required to produce the large vector volumes used in this study resulted in a vector titre that was 10-fold lower than previously used in mouse studies. Furthermore, the long tracheal length and the considerably larger than expected ratio between lung volume and body weight/size meant that animals did not receive sufficient gene vector. Overall, this suggests that a linear scale up by weight from previous mouse studies was insufficient. Results from the analysis of p24 presence in the blood serum also support the notion that the vector dose was insufficient.

The intubation tube was placed with the opening mid-way between the epiglottis and the carina so that the bulk of the pre-treatment and vector dose would be delivered to the distal portion of the trachea and lung. However, histological examination showed that there was LacZ expression along the length of the trachea, indicating significant retrograde transduction had occurred (as observed in mouse fluid-dosing studies [20]). Moreover, this would affect the volume reaching the distal trachea, lower conducting airways, and lung parenchyma.

Since the ferret trachea is very long (9 cm) and the lung volume is approximately three-fold larger than predicted allometrically [12, 19], it is likely that the delivered pre-treatment and vector doses were not sufficient to produce robust gene expression in the airways. The pre-treatment and vector volumes may not have acted on the same regions of airway epithelium, further reducing the effectiveness of the vector.

Although the bulk of the delivered doses may have been retained in the trachea, the level of tracheal transduction was still lower than expected. It is conceivable that the prone positioning resulted in retention of LPC within the trachea that may have subsequently reduced the viability of the LV vector. Furthermore, in this orientation vector dose could be more readily lost via fluid clearance and swallowing.

## Conclusion

Despite the low levels of gene expression the outcomes confirm that the airways of the ferret can be transduced utilizing our gene delivery protocol employing LPC pre-treatment and a HIV-1 VSV-G pseudotyped lentiviral vector. Ciliated and basal cells types can be transduced, potentially providing a basis for both immediate and sustained transgene expression, respectively. This study has also revealed unique aspects of ferret conducting airways and alveolar space anatomy that may have affected the level of gene expression achieved. Ensuring adequate pre-treatment and vector volume, maximising the vector titre, attention to animal orientation during dosing, and determining the influence of LPC are specific factors to be addressed when embarking on future studies in normal and CF ferrets. With optimal dose volumes and vector titre for the ferret lung anatomy the levels of gene transfer are likely to approach those in previous mouse studies and provide effective protocols for future use of lentiviral CFTR gene transfer studies in CF ferrets.

## Abbreviations

CF: Cystic fibrosis
CFTR: Cystic fibrosis transmembrane conductance regulator
LPC: Lysophosphatidylcholine
LV: Lentiviral
PBS: Phosphate buffered saline.
